# Functional network connectivity is altered in patients with upper limb somatosensory impairments in the acute phase post stroke: A cross-sectional study

**DOI:** 10.1371/journal.pone.0205693

**Published:** 2018-10-12

**Authors:** Nele De Bruyn, Sarah Meyer, Simon S. Kessner, Bea Essers, Bastian Cheng, Götz Thomalla, Andre Peeters, Stefan Sunaert, Thierry Duprez, Vincent Thijs, Hilde Feys, Kaat Alaerts, Geert Verheyden

**Affiliations:** 1 KU Leuven—University of Leuven, Department of Rehabilitation Sciences, Leuven, Belgium; 2 University Medical Center Hamburg-Eppendorf, Department of Neurology, Hamburg, Germany; 3 Cliniques Universitaires Saint-Luc, Department of Neurology, Brussels, Belgium; 4 KU Leuven—University of Leuven, Department of Imaging and Pathology, Leuven, Belgium; 5 University Hospitals Leuven, Department of Radiology, Leuven, Belgium; 6 Cliniques Universitaires Saint-Luc, Department of Radiology, Brussels, Belgium; 7 University of Melbourne, Florey Institute of Neuroscience and Mental Health, Victoria, Australia; 8 University of Melbourne, Department of Neurology, Austin Health, Victoria, Australia; University of Texas at Austin, UNITED STATES

## Abstract

**Background:**

Aberrant functional connectivity in brain networks associated with motor impairment after stroke is well described, but little is known about the association with somatosensory impairments.

**Aim:**

The objective of this cross-sectional observational study was to investigate the relationship between brain functional connectivity and severity of somatosensory impairments in the upper limb in the acute phase post stroke.

**Methods:**

Nineteen first-ever stroke patients underwent resting-state functional magnetic resonance imaging (rs-fMRI) and a standardized clinical somatosensory profile assessment (exteroception and higher cortical somatosensation) in the first week post stroke. Integrity of inter- and intrahemispheric (ipsilesional and contralesional) functional connectivity of the somatosensory network was assessed between patients with severe (Em-NSA< 13/32) and mild to moderate (Em-NSA> 13/32) somatosensory impairments.

**Results:**

Patients with severe somatosensory impairments displayed significantly lower functional connectivity indices in terms of interhemispheric (p = 0.001) and ipsilesional intrahemispheric (p = 0.035) connectivity compared to mildly to moderately impaired patients. Significant associations were found between the perceptual threshold of touch assessment and interhemispheric (r = -0.63) and ipsilesional (r = -0.51) network indices. Additional significant associations were found between the index of interhemispheric connectivity and light touch (r = 0.55) and stereognosis (r = 0.64) evaluation.

**Conclusion:**

Patients with more severe somatosensory impairments have lower inter- and ipsilesional intrahemispheric connectivity of the somatosensory network. Lower connectivity indices are related to more impaired exteroception and higher cortical somatosensation. This study highlights the importance of network integrity in terms of inter- and ipsilesional intrahemispheric connectivity for somatosensory function. Further research is needed investigating the effect of therapy on the re-establishment of these networks.

## Introduction

Modern brain imaging techniques such as resting-state functional magnetic resonance imaging (rs-fMRI) allow to assess intrinsic network connectivity by exploring spontaneous fluctuations of the blood oxygen level dependent (BOLD) signal in the low-frequency range while people are at rest in the scanner [1]. In the healthy human brain, distinct functionally relevant ‘resting-state’ networks have consistently been identified across individuals with a high reproducibility across time points and age ranges [2]. In seminal work by Biswal et al. [3], a first description of the resting-state sensorimotor network was provided, and later also other functionally relevant networks were described, including the default mode network and the attention network [4, 5].

In patient populations, alterations in networks have been associated with changes in behavioral performance [6–10]. In stroke patients, the presence of neglect has been associated with decreased functional connectivity in the dorsal attention network [8, 11–13]. Similar results are reported for the association with motor performance [11, 14]. Alterations in functional connectivity related to behavioral deficits have been described for different stages post stroke. In general, overall decreased functional connectivity is found in the acute phase post stroke [14, 15], where a gradual increase towards normalization during subacute and chronic phase is reported to develop parallel with recovery of performance [16, 17].

Network changes are reported for inter- and intrahemispheric functional connectivity. In post stroke aphasia, increased interhemispheric and contralesional intrahemispheric functional connectivity and reduced ipsilesional intrahemispheric connectivity was associated with poorer language skills [18]. With regard to motor function, decreased interhemispheric and increased contralesional intrahemispheric functional connectivity are described [14]. The role of the contralesional hemisphere has been described as changing over time, starting from hyperactive due to a lack of inhibitory activity of the ipsilesional hemisphere towards a more supportive role with decrease of functional connectivity towards normalization [19].

Since somatosensory and motor function are closely related concepts [20], and the sensorimotor network is described for both functions [3], similar results are expected for somatosensory function. However, evidence is limited. Chen et al [21] described increased functional connectivity between contralesional mid temporal gyrus and the stroke area in patients with thalamic stroke. Further, only two papers investigated the effect of a somatosensory intervention on functional connectivity. The first study, investigating the effect of passive proprioceptive wrist training on motor function, indicated an increase in functional connectivity in the inferior parietal cortex [22]. The other study, investigating the effect of sensorimotor therapy on somatosensory and motor function, reported a small increase in activated brain volume of the deactivated area after four weeks of therapy. However, they did not found any changes in connectivity of the superior thalamic radiation [23]. Finally, only one study investigated functional connectivity of the somatosensory network in subacute ischemic stroke patients [24]. Particularly, they explored the association between performance on a touch discrimination task and changes in resting state functional connectivity one and six months post stroke. Compared to healthy controls, interhemispheric functional connectivity between sensorimotor areas, bilateral primary somatosensory cortices and bilateral thalamus, was shown to be reduced at one-month post stroke. Additionally, recovery of touch discrimination performance was found to be strongly associated (R^2^ = 0.72) with increased contralesional intrahemispheric functional connectivity between S2 and interparietal cortex and midtemporal gyrus at 6 months compared to 1 months.

While the latter study provided initial insights in neural alterations in the *subacute* phase post stroke, it is currently unclear whether similar neural alterations in inter- and intrahemispheric connectivity of the somatosensory network are already evident in the *acute* phase post stroke. Furthermore, insights in differences in functional connectivity between more severely and mildly to moderately impaired patients are lacking. The study of Bannister et al. [24] focused on one somatosensory modality (touch discrimination) during the rest phase in between the task fMRI scanning periods. Therefore, it can be noticed that this brain-behavior association is not yet explored in the acute phase, and specifically how impairments in different modalities of somatosensory functioning including light touch, pressure, but also sharp-dull discrimination and stereognosis, are reflected in changes in resting-state networks. To fill this gap, the present study aims to investigate resting-state functional connectivity in the acute phase post stroke and to identify how changes in inter- and intrahemispheric network connectivity of several key somatosensory areas relate to behavioral performance and to severity of impairments in several modalities of somatosensory function.

First, and in accordance to prior observations of reductions in interhemispheric connectivity post-stroke, we investigated whether reduced interhemispheric connectivity is evident in the somatosensory network in the acute phase post stroke and particularly, whether the extent of reduced interhemispheric connectivity relates to behavioral somatosensory impairments. In terms of intrahemispheric connectivity, Bannister et al showed that the intrahemispheric connectivity in the contralesional hemisphere was more strongly associated with improved somatosensory function at six months compared to one month post stroke [24]. Here, we aimed to explore whether levels of intrahemispheric connectivity in the acute phase post-stroke are associated with the extent of somatosensory deficits. For both hemispheres separately, we expected a decrease of functional connectivity that would be associated with impaired somatosensory function, as seen in motor function [25].

## Materials and methods

### 2.1 Subjects and setting

The study was carried out in accordance with the latest version of the Declaration of Helsinki and approval from the ethics committee of both university hospitals was obtained. Written informed consent was provided by all patients prior to participation. For this cross-sectional observational study, 27 patients with upper limb sensorimotor impairments after stroke were recruited from two acute stroke units in Belgium; University Hospitals Leuven and University Hospital St-Luc Brussels; within the first four to seven days post stroke. Inclusion criteria for participation in the study were: confirmed first-ever stroke based on the definition of the World Health Organization (WHO MONICA project principal investigators) [26], both subcortical and cortical lesions, minimum age of 18 years, sufficient cooperation to perform the assessment and a motor and/or somatosensory deficit in the upper limb. The presence of a motor impairment was defined as a score of <60 out of 66 on the upper extremity part of the Fugl-Meyer motor assessment [27], whereas a somatosensory impairment was defined as a score of ≥1 out of 2 on item 8 of the National Institutes of Health Stroke Scale (NIHSS) [28]. Patients with musculoskeletal and/or other neurological impairments such as previous stroke, head injury or multiple sclerosis, as well as patients with stroke-like symptoms caused by subdural hematoma, tumor, encephalitis or trauma, were excluded from this study. Other exclusion criteria were a pre-stroke Barthel index score <95 out of 100 [29] and severe communication, cognitive or language deficits. Note that eight out of the initial 27 patients were not included in the final analyses due to excessive in-scanner head motion (mean frame-wise displacement exceeding 0.5 mm). As such, final analyses were performed on a total of 19 patients.

### 2.2 Testing protocol

Patients were assessed in one single test session including a behavioral assessment and an MRI brain imaging protocol in the acute phase post stroke (i.e., between the fourth and seventh day post stroke).

#### 2.2.1 Behavioral assessment

Somatosensory function of the affected upper limb was assessed using the Erasmus MC modified (revised) Nottingham Sensory Assessment (Em-NSA), stereognosis subscale of the original NSA, two-point discrimination test (2PD), and perceptual threshold of touch (PTT). To assure standardization, behavioral assessment was performed by one trained researcher. A detailed description of the behavioral assessment protocol can be found in our previously published work [30]. All behavioral assessment tools are reliable and valid measurements [31–34].

The Em-NSA comprises four subscales: light touch, pressure, pinprick, and sharp-dull discrimination [34]. A total score between 0 and 8 was obtained for each modality. A cut-off score of <7 out of 8 for each subscale was defined as impaired function. A total score between 0 and 32 was calculated by summing all subscores. The original NSA was used to assess the stereognosis function [31] by providing 11 commonly-used objects in the affected hand leading to a total score of 22. The cut-off score to define a stereognosis deficit was <19 out of 22 [35]. Two-point discrimination [32] tests the ability to detect two different stimuli that are simultaneously applied at the fingertip of the index finger. A cut-off score of >5mm was defined as impaired two-point discrimination function [36]. Finally, the perceptual threshold of touch [33] assesses the minimal level of detection of a touch stimulus [33] by applying transcutaneous electrical nerve stimulation (TENS) with a CEFAR Primo Pro (cefar medical AB, Sweden). The scores were compared to age- and gender-matched norm values [37].

#### 2.2.2 Statistical analysis of the behavioral assessments

Baseline characteristics and behavioral assessments were analyzed using SPSS version 23. Descriptive analyses were performed using median with interquartile range (IQR) and frequencies with percentages, as appropriate. The prevalence of different somatosensory deficits such as light touch or sharp-dull discrimination impairment were calculated using frequencies with percentages. Therefore, the different somatosensory variables were dichotomized according to the presence of a deficit or normal functioning, based on the above-mentioned pre-defined cut-off values.

#### 2.2.3 Brain imaging data acquisition

Anatomical and resting-state fMRI images of all patients were acquired on the same 3.0 Tesla Philips MR scanner (Best, The Netherlands) with an 8-channel phased-array head coil. Scan sessions started with the acquisition of the anatomical scan, followed by the resting-state fMRI scan. Anatomical images were acquired using fluid-attenuated inversion recovery imaging (FLAIR) with the following parameter settings: echo time (ET) = 350ms, repetition time (TR) = 4800ms, inversion time = 1650ms, field of view (FOV) = 250x250mm^2^, slice thickness = 1.12mm and interslice gap = 0.56mm. The resting state fMRI images consist of a total of 30 parallel transverse orientated slices of 4mm thickness with no interslice gap. Parameter settings were: TE = 33ms, flip angle 90°, TR = 1700ms, FOV = 230mm, matrix = 64x64, duration 7 min. During scanning, participants were lying in supine position with eyes closed and were instructed to think of nothing in particular and to not fall asleep. Based on an informal survey, no patients have fallen asleep during scanning.

#### 2.2.4 Preprocessing of resting state data

Co-registration and delineation of the lesions, white matter and cerebrospinal fluid were acquired form the T1-structural images to implement as confounding factor in the pre-processing of the resting state fMRI data. The process of lesion segmentation was reported in detail in a previous study of our research group [38]. In short, delineation of the lesion was manually performed in several steps by an experienced rater (SK) to ensure accuracy of the lesion delineation. Linear transformation was applied to transform the individual FLAIR data and lesion segmentation into a standardized brain template in MNI space (Montreal Neurological Institute).

Preprocessing of resting state fMRI images was performed with SPM-8 (Wellcome Department of Imaging Neuroscience, London, UK) and the CONN functional connectivity toolbox [39] both implemented in Matlab R2008a (Mathworks). Preprocessing of the data included slice timing correction through interpolation to the middle slices (reference = 17). Then, the functional images were spatially realigned and the anatomical T1-image was co-registered to the mean functional image to map functional information into anatomical space. Anatomical and functional images were then normalized to the standard EPI-template of the Montreal Neurological Institute (MNI-152), resampled into 3-mm isotropic voxels and smoothed (Gaussian smoothing; isotropic 5mm full-width-at-half-maximum).

Realignment parameters were included as regressors of no interest. The CompCor strategy [40] was applied in the CONN functional connectivity toolbox to correct for confounding factors in white matter and cerebrospinal fluid. Then, the residual time series of the resting state images were band-pass filtered (0.009< f <0.08Hz). Since even micromovements can already influence intrinsic functional connectivity [41, 42], mean frame-wise displacement (FD) was calculated for each patient and included as a nuisance covariate in the group analyses. Note that patients with excessive head motion, defined as mean FD scores exceeding 0.5 were not included in the final analyses (8 out of the initial 27 patients were excluded). Additionally, to account for lesions, region-to-region functional connectivity analyses were performed with and without regions overlapping with lesion-delineated maps.

#### 2.2.5 Resting-state connectivity analysis

To delineate the functional network relevant for somatosensory processing, twelve bilateral Regions of Interest (ROIs) (6 left, 6 right) were adopted from a task-based fMRI study of Chang and colleagues, exploring sensory processing in nine healthy participants[43]. The central coordinates of the predefined spherical ROIs (6 mm diameter) are reported in Table 1.

**Table 1:** MNI coordinates of the twelve regions-of-interests (ROIs) of the somatosensory processing network adopted to explore ROI-to-ROI resting-state functional connectivity.

		LEFT			RIGHT		
		x	y	z	x	y	z
Primary sensorimotor cortex	**SM1**	-63	-23	47	54	-23	44
Superior parietal cortex	**SPC**	-29	-48	64	17	-54	50
Supplementary motor cortex	**SMA**	-4	-10	57	7	-9	64
Inferior parietal cortex	**IPC**	-54	-43	31	56	-36	30
Insula	**INS**	-28	-11	20	44	-19	12
Cerebellum	**CER**	-23	-63	-37	18	-56	-25

According to Chang et al. 2009. MNI: Montreal Neurological Institute

**Fig 1:**
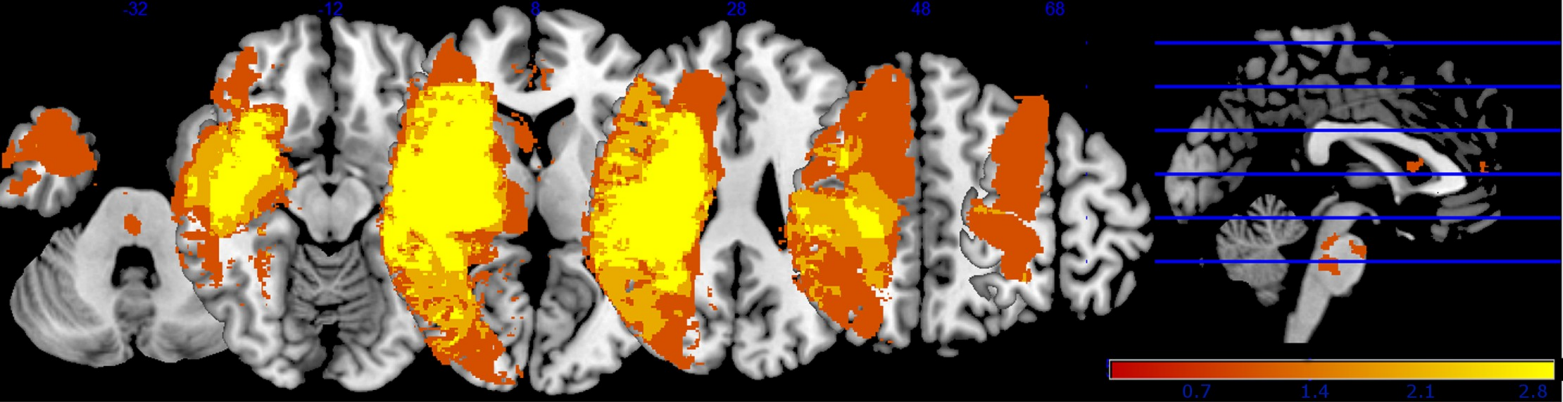
Group lesion overlay image. Fig 1 displays the lesion location of all patients. All lesions were flipped to the right site. Color indicates the lesion locations from red (not lesioned in any patient) to yellow (lesioned in most patients).

For each participant, we extracted the mean time series by averaging across all voxels in each ROI. We then computed bivariate correlation coefficients for each pair of ROIs. In the primary analysis, only ROI-to-ROI correlation coefficients were included for connections that showed no overlap with the predefined lesion [11]. MRIcron (software package version 1, 2015) was used to identify any overlap between the standardized lesion delineations and any of the predefined regions-of-interest (ROIs). Across patients, ROI-to-ROI correlation coefficients were therefore not included in the primary analysis for 26 connections due to overlap with the delineated lesions (average of 2.25 connections per patient). A group lesion overlay image is provided in Fig 1. (see S1 Table for detailed list of connections that showed overlap with the lesions of each patient). Note that secondary analyses were performed on the entire set of ROI-to-ROI correlation coefficients (i.e., also including the 26 connections that originated from ROIs in the predefined lesions) to determine the robustness of the main results of the primary analysis.

The resultant ROI-to-ROI correlation coefficients were Fisher z-transformed and extracted to perform group-level analyses and brain-to-behaviour correlation analyses in SPSS version 23.

Related to our a-priori hypotheses on alterations in intra- and interhemispheric connectivity, the following indices of network connectivity were calculated:

- To obtain an index of intrahemispheric network connectivity of the ipsilesional and contralesional hemisphere, ROI-to-ROI correlation coefficients of all eligible connections (i.e., outside the lesion) in the affected (6 x 6 connections) and unaffected hemisphere (6 x 6 connections), respectively, were averaged separately for each participant.
- To obtain an index of interhemispheric network connectivity, ROI-to-ROI correlation coefficients between homologue ROIs (total of 6 interhemispheric connections) were averaged for each participant.

Next, for all indices of functional network connectivity, Shapiro-Wilk normality tests were performed to assess normality of distribution. Normality was not confirmed for the functional connectivity index of the ipsilesional intrahemispheric network due to one extreme outlier. Particularly, one patient showed an extreme ipsilesional intrahemispheric connectivity score (defined as smaller than Q1 ± 3(Q3-Q1), with Q1 and Q3 being the first and third quartile). All primary analyses were performed after removal of the outlier data point, however, for completeness; secondary analyses were performed with inclusion of the outlier to assess the robustness of the primary results. Two groups were created based on the median value of the total score on Em-NSA, to explore whether indices of functional network connectivity were significantly different between patients with severe somatosensory impairments (n = 9) (Em-NSA score of <13 out of 32) and patients with mild to moderate somatosensory impairments (n = 10) (Em-NSA ≥13 out of 32). First, mean and standard deviations of functional connectivity values of each index were calculated for the total group and for each subgroup (mild to moderate and severe). Further, to explore differences in functional connectivity between subgroups, a general linear model was constructed with the index of network connectivity as dependent variable; ‘group’ (severe, mild to moderate) as between-subject factor, and age, centre (stroke unit where data were collected) and mean framewise displacement (FD) as covariates of no interest (nuisance regressors). Additionally, to investigate potential brain-behavioral associations between the indices of functional network connectivity and scores of the behavioral somatosensory assessments, non-parametric partial correlations were calculated also including age, center and mean FD as nuisance regressors. Non-parametric spearman rho correlation tests were adopted due to ordinal scoring of the behavioral somatosensory measures. Brain-behavior associations were assessed with the following behavioral somatosensory assessments; (i) light touch, (ii) pressure, (iii) pinprick, (iv) sharp-dull discrimination, (v) stereognosis, (vi) two-point discrimination, and (vii) perceptual threshold of touch. Residuals of the partial correlations were extracted and plotted against the behavioral assessments, when significant.

## Results

### 3.1 Patient characteristics and behavioral outcome

Patient characteristics of the 19 included participants are presented in Table 2. Eight males (42%) and 11 females (58%) were assessed at a median of 6 days after stroke (IQR 5–7). The age ranged between 27 and 92 years with a median of 77 years (IQR 66–86). The majority (N = 15; 79%) presented with a lesion in the right hemisphere, in 14 patients (74%) the lesion was caused by ischemic stroke and 5 patients (26%) had hemorrhagic stroke. Most patients (N = 16, 84%) had combined cortical and subcortical lesions, 1 patient (5%) had a pure sub-cortical lesion in the basal ganglia and posterior limb of internal capsule and 2 patients (11%) had lesions in the brainstem. (A detailed overview of lesion location is provided in S2 Table). The scores on the NIHSS ranged between 5 and 18 with a median score of 9 (IQR 6–15), indicating moderate to severe stroke severity. In addition, generally poor somatosensory function in the upper limb was found with a median score of 13 out of 32 (IQR 0–29) on the Em-NSA scale for somatosensory function. Three patients (16%) showed no impairment in any of the somatosensory subscales.

**Table 2:** Patient characteristics (n = 19).

		Total n = 19	Mild to moderate n = 10	Severe n = 9
Age stroke onset, years: median (IQR)		76.6 (66.1–85.8)	76.8 (75.5–81.8)	74.6 (50.3–87.5)
Gender: n (%)				
	Male	8 (42.1)	5 (50)	3 (33.3)
	Female	11 (57.9)	5 (50)	6 (66.7)
Centre: n (%)				
	University Hospitals Leuven	7 (36.8)	6 (60)	1 (11.1)
	Cliniques Universitaires Saint-Luc	12 (63.2)	4 (40)	8 (88.9)
Days after stroke: median (IQR)		6 (5–7)	6 (5.8–7)	6 (5–7)
Lateralisation: n (%)				
	Right hemisphere lesion	15 (78.9)	8 (80)	2 (22.2)
	Left hemisphere lesion	4 (21.1)	2 (20)	7 (77.8)
Type of stroke: n (%)				
	Ischemia	14 (73.7)	7 (70)	7 (77.8)
	Haemorrhage	5 (26.3)	3 (30)	2 (22.2)
Hand dominance: n (%)				
	Left	1 (5.3)	1 (10)	0 (0)
	Right	18 (94.7)	9 (90)	9 (100)
National Institutes of Health Stroke Scale (/42): median (IQR)		9 (6–15)	8 (6–11.5)	13 (7–17)
Fugl-Meyer, upper extremity (/66): median (IQR)		4 (2–55)	7 (2–55.5)	4 (3–51.5)
Em-NSA- total (/32): median (IQR)		13 (0–29)	29 (18.8–32)	0 (0–5)
Em-NSA- light touch (/8): median (IQR)		5 (0–7)	6.5 (6–8)	0 (0–0)
Em-NSA- pressure (/8): median (IQR)		4 (0–8)	8 (5–8)	0 (0–3)
Em-NSA- pinprick (/8): median (IQR)		4 (0–8)	8 (8–8)	0 (0–2)
Em-NSA- sharp-dull discrimination (/8): median (IQR)		0 (0–7)	6.5 (1.5–8)	0 (0–0)
NSA- stereognosis (/22): median (IQR)		0 (0–19)	17 (1.5–21)	0 (0–0)
Two-point discrimination (/16): median (IQR)		16 (16–16)	16 (16–16)	16 (4.8–16)
Perceptual threshold of touch (/11) median (IQR) (n = 18):		6 (4.4–11)	11 (11–11)	4.8 (3.9–5.5)
Functional connectivity- Interhemispheric network: mean (SD)		0.336 (0.125)	0.419 (0.093)	0.245 (0.096)
Functional connectivity- Ipsilesional intrahemispheric network: mean (SD)		0.065 (0.083)	0.102 (0.089)	0.018 (0.045)
Functional connectivity- Contralesional intrahemispheric network: mean (SD)		0.082 (0.081)	0.091 (0.090)	0.071(0.073)

Table 2 provides the patient characteristics for the total group (left) and mild to moderate (middle) or severe (right) separately. Functional connectivity is displayed with z-transformed r-values for the different indices without values of connections containing a ROI located in the lesion area. Ipsilesional intrahemispheric network consist of connectivity values of all persons without the outlier.

IQR: interquartile rage; Em-NSA: Erasmus MC modified (revised) Nottingham sensory assessment; NSA: Nottingham Sensory Assessment SD: standard deviation.

**Fig 2:**
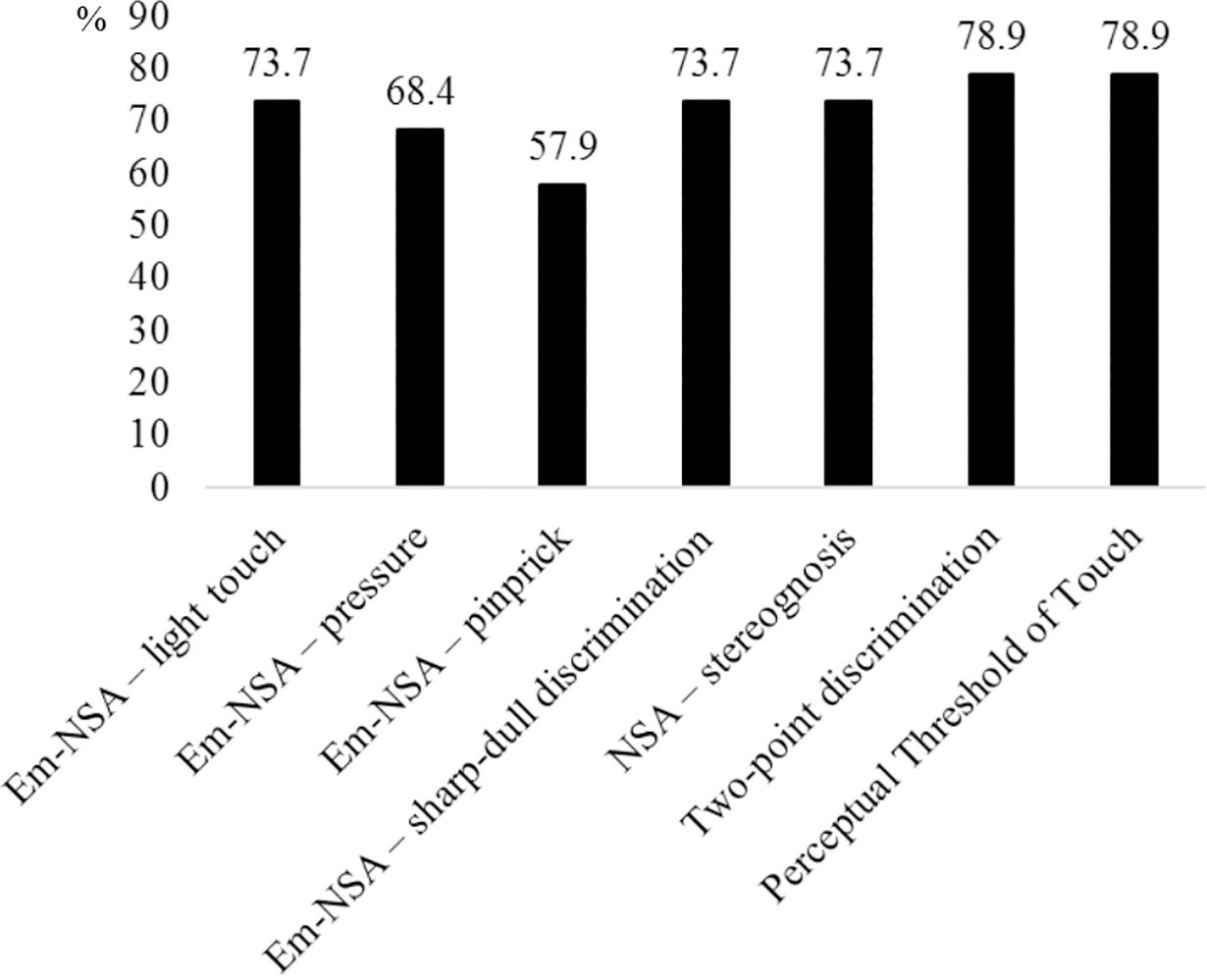
Prevalence of somatosensory deficits. Fig 2 displays the prevalence of somatosensory deficits for each subscale of the Em-NSA, Two-poindiscrimination test and Peceptual Threshold of Touch. Em-NSA: Erasmus MC modified (revised) Nottingham sensory assessment; NSA: Nottingham sensory assessment (gray scale, 1.5 colllum).

Fig 2 shows the prevalence of different somatosensory deficits. Based on the scores of the Em-NSA subscales, a total of 14 (74%) patients had deficits in light touch, 13 (68%) in pressure, 11 (58%) in pinprick, and 14 (74%) in sharp-dull discrimination. Both the perceptual threshold of touch, and the two-point discrimination were impaired in 15 (79%) patients. Finally, 14 patients (74%) experienced a stereognosis deficit.

### 3.2 Network connectivity

#### 3.2.1 Group differences in somatosensory network connectivity between patients with severe and mild to moderate somatosensory impairments

**Fig 3:**
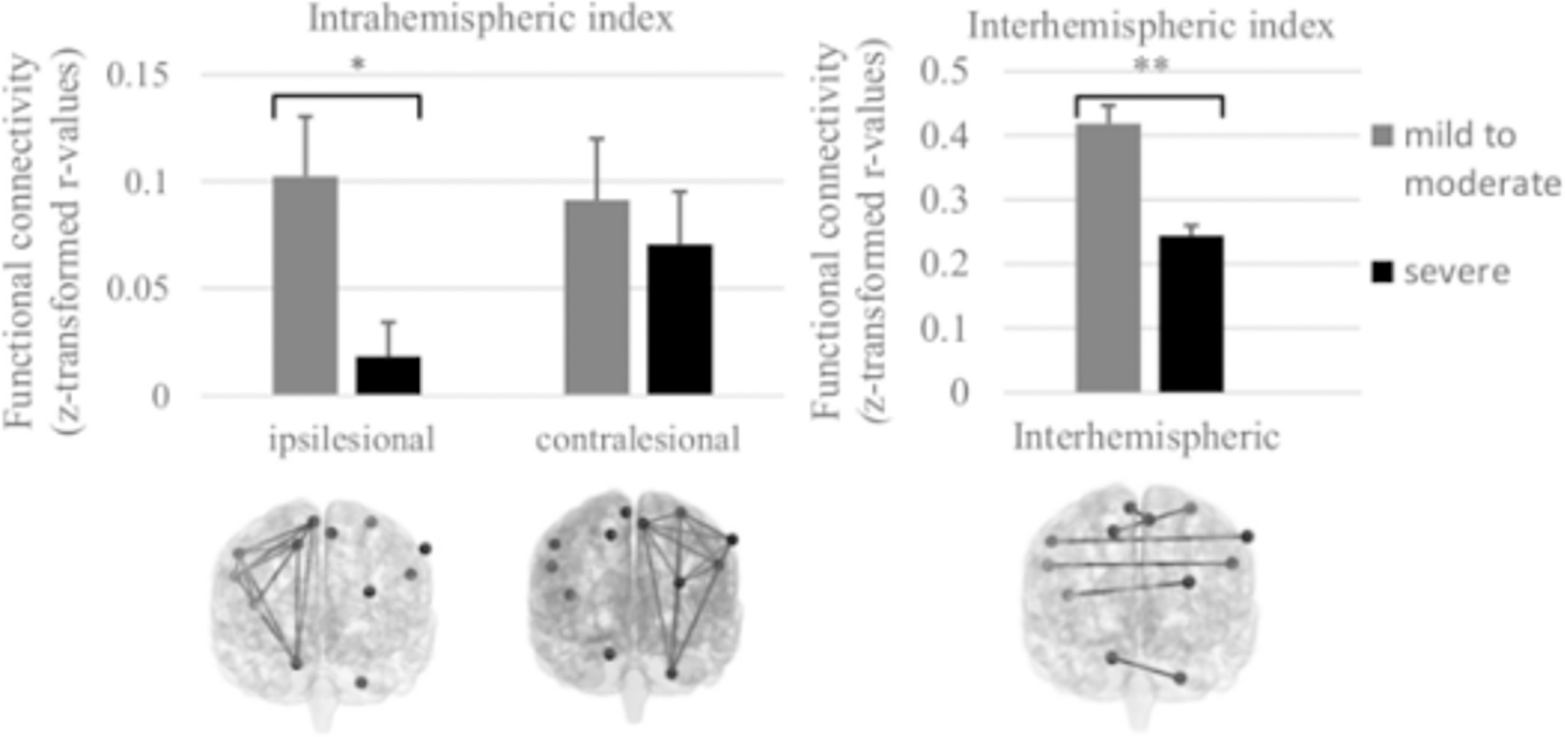
Group differences in somatosensory network connectivity between patients with severe and mild to moderate somatosensory impairment. Fig 3 shows the differences in functional connectivity between subgroups, investigated by a general linear model with the index of network connectivity as dependent variable; ‘group’ (severe, mild to moderate) as between-subject factor, and age, centre (stroke unit where data were collected) and mean framewise displacement (FD) as covariates of no interest (nuisance regressors). Patients with severe somatosensory impairments (Em-NSA <13/32) displayed significantly lower network intrinsic connectivity in the ipsilesional intrahemispheric and interhemispheric network compared to patients with mild to moderate somatosensory impairments (Em-NSA ≥13/32). Networks are displayed in frontal plane in back view; Error bars display the Standard Error of mean; *p< 0.05; ** p<0.01; ***p<0.001 (gray scale, 2-collum fitting).

Patients with severe somatosensory impairments (n = 9) showed significantly lower intrinsic functional connectivity compared to patients with mild to moderate somatosensory impairments (n = 10) in terms of (i) intrahemispheric connectivity of the ipsilesional hemisphere (F(1,18) = 5.667; p = 0.035); and (ii) interhemispheric connectivity between homologue regions (F(1,19) = 17.088; p = 0.001). Group differences were also found in terms of intrahemispheric connectivity of the contralesional hemisphere; lower connectivity scores were revealed in the patient group with severe compared to mild impairment, albeit not significant (F(1,19) = 2.011; p = 0.180) (Fig 3).

When analyses were performed across all connections (i.e., ‘whole-network’ functional connectivity combining all inter- and intra-hemispheric connections (12 x 12 connections)) a significant reduction in functional connectivity of the somatosensory network was revealed indicating lower connectivity in the severe, compared to the mild affected patient group (F(1,19) = 9.945; p = 0.008).

#### 3.2.2 Associations between connectivity of the somatosensory network and somatosensory impairments

Brain-behavioral relationships were assessed between the indices of network connectivity and behavioral assessments of somatosensory impairment. Similar to the categorical group-related analysis, a general pattern emerged, indicating that—on a dimensional scale—patients with more severe somatosensory impairments showed reduced functional connectivity of the somatosensory network. For intrahemispheric connectivity of the contralesional hemisphere, low, non-significant associations were found (r = 0.23–0.42).

**Fig 4:**
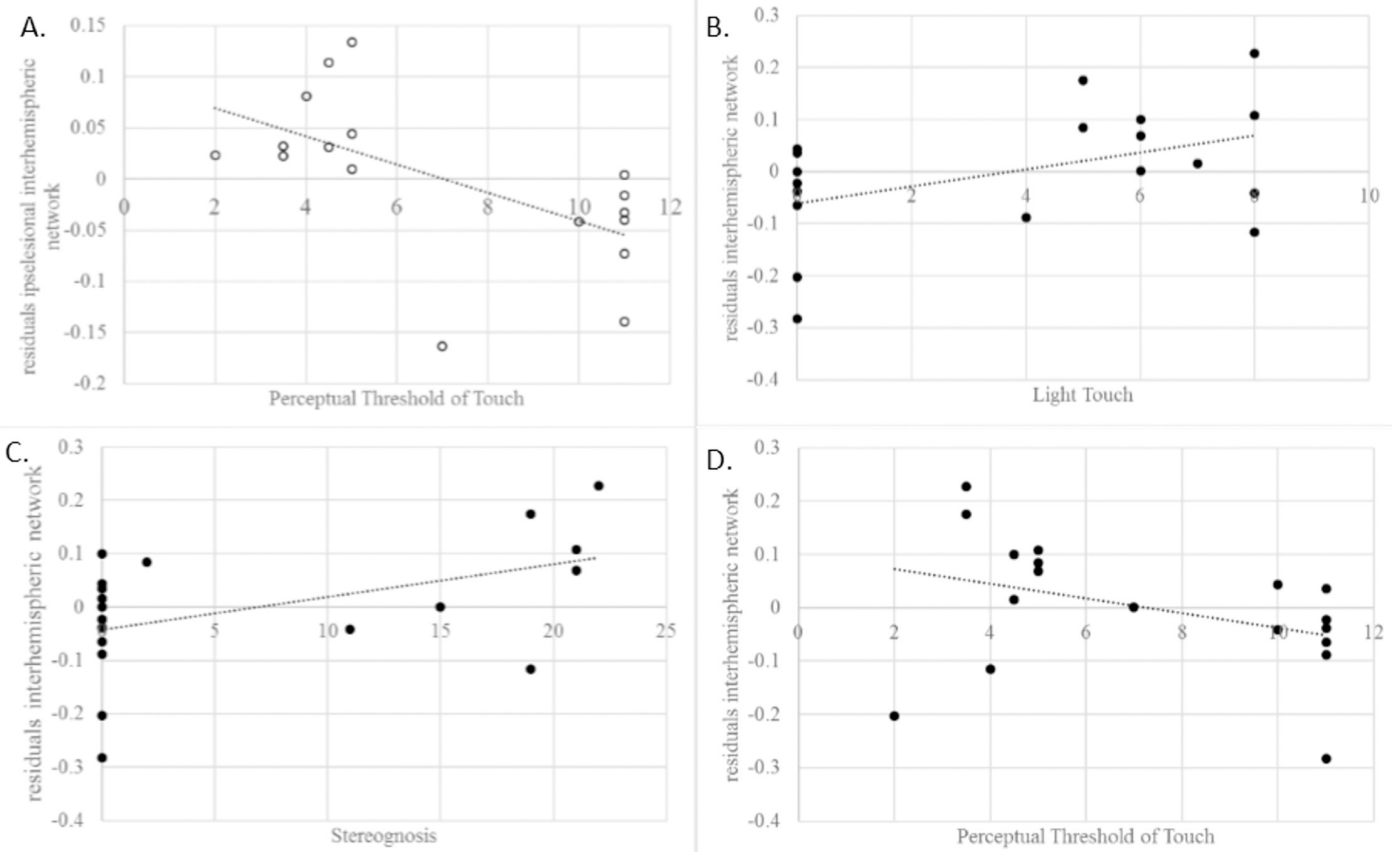
Brain-behaviour associations between specific somatosensory modalities. Correlation plots between behavioural assessment and residuals of the partial correlations with the index inter- or intrahemispheric functional connectivity. Correlation plot between the perceptual threshold of touch and ipsilesional intrahemispheric network (A) and interhemispheric network (B); correlation plot between interhemispheric network and light touch (C) and stereognosis (D). (gray scale; 2-collum fitting).

As displayed in Table 3, it can be seen that reductions in (i) intrinsic functional connectivity of the ipsilesional hemisphere (r = -0.69; p<0.01), and (ii) interhemispheric connectivity (r = -0.51; p<0.05) were significantly related to somatosensory impairment—in terms of a higher perceptual threshold of touch. Furthermore, reductions in interhemispheric connectivity were also significantly associated with somatosensory impairments in terms of light touch (r = 0.55; p<0.05) and stereognosis (r = 0.64; p<0.01), indicating that patients with more severe impairments showed larger reductions in interhemispheric connectivity. Scatterplots were created to visualize the individual brain-behavior correlations. (Fig 4).

**Table 3:** Partial correlations (non-parametric) between network indices and somatosensory modalities.

	Light Touch	Pressure	Pinprick	Perceptual Threshold of Touch^1^	Sharp/Dull discrimination	Stereognosis	Two-point discrimination
Ipsilesional intrahemispheric network	0.48	0.40	0.31	-0.69**	0.19	0.33	-0.35
Contralesional intrahemispheric network^1^	0.04	0.09	0.22	-0.42^2^	0.20	0.23	0.01
Interhemispheric network	0.55*	0.45	0.48	-0.51*	0.42	0.64**	-0.28

** Correlation is significant at the 0.01 level (2-tailed)

* Correlation is significant at the 0.05 level (2-tailed); Control variables: age, centre, mean framewise displacement; df = 14

^1^df = 13

^2^df = 12

Note however that the dimensional brain-behavior association with perceptual threshold of touch seemed to be largely driven by a categorical clustering of patients with ‘more severe’ or ‘more mild/moderate’ impairment on this behavioral score. Indeed, the dimensional relationship between perceptual threshold of touch and the indices of ipsilesional intrahemispheric connectivity or interhemispheric functional connectivity failed to reach significance when the factor ‘group’ (severe vs mild/moderate) was inserted as an additional nuisance regressor, indicating that the majority of the observed inter-individual variance in functional connectivity was already explained by the categorization of the patients in ‘severe’ versus ‘mild to moderate’ subdivisions.

#### 3.2.3 Secondary analyses

Secondary analyses were performed to determine whether the main findings of the primary analyses were replicated when connectivity analyses are performed on the entire set of ROI-to-ROI correlation values (i.e., also including connections that originated from ROIs in the predefined lesions). Overall, a similar pattern of results was shown, indicating that more severe somatosensory impairment was significantly associated with reductions in functional connectivity of the somatosensory network (most pronounced for interhemispheric connectivity and intrahemispheric connectivity of the ipsilesional hemisphere). Furthermore, in the primary analysis, the data point of one ‘outlier’ patient was excluded in the analysis of intrahemispheric connectivity of the ipsilesional hemisphere. Secondary analysis with inclusion of this data point replicated all primary results, i.e., indicating that reduced ipsilesional connectivity was associated with more severe somatosensory impairment.

## Discussion

The aim of this study was to investigate functional network connectivity at rest between different key brain regions of the somatosensory network in the acute phase post stroke and the association with resulting upper limb somatosensory impairments. As hypothesized, patients with mild to moderate somatosensory impairments showed significant higher functional connectivity compared to patients with severe somatosensory impairments. This difference in functional connectivity was found for the indices of interhemispheric and ipsilesional intrahemispheric functional connectivity but not for the index of contralesional intrahemispheric functional connectivity. Furthermore, as stated a priori, reduced levels of inter- and intra-hemispheric functional connectivity of the somatosensory network were associated with more severe upper limb somatosensory impairments.

### 4.1 Altered functional connectivity

In general, decreased functional connectivity after stroke is associated with impaired function [6, 16, 44–48]. Similarly, higher functional connectivity is associated with better function [16, 46] and our results contribute to this field of knowledge in relation to upper limb somatosensory impairments. This association with function is observed in healthy and stroke subjects. In healthy subjects, Haag et al. [49] investigated the association between functional connectivity and performance on a two-point discrimination test and found that higher BOLD amplitudes and stronger regional homogeneity of the voxels in the hand region in the primary somatosensory cortex were associated with lower, and therefore better, two-point discrimination thresholds. This is in line with our findings of overall lower functional connectivity values in patients with severe somatosensory impairments compared to patients with mild to moderate impairments. In stroke animal and human subjects, longitudinal studies described a decreased functional connectivity in the acute phase after stroke and an increase towards normalisation of functional connectivity over time, associated with recovery of function [16, 17, 50]. Thus, severity of impairment seems to be associated with the level of functional connectivity and the latter could potentially be a biomarker for recovery [51].

#### 4.1.1 Decreased interhemispheric functional connectivity

In literature, decreased interhemispheric functional connectivity is described in rats and humans after stroke to be associated with impaired sensorimotor function [6, 47, 50, 52], and our study adds to this concept for the domain of arm and hand somatosensory deficits. Carter et al. [48] presented an association between a disruption in interhemispheric functional connectivity of the sensorimotor network and upper extremity motor impairments in the acute phase post stroke. Furthermore, Bannister et al. [24] explored the association of alterations in functional connectivity with somatosensory impairments; a relationship was reported between decreased interhemispheric functional connectivity between ipsilesional and contralesional primary somatosensory cortices and thalamus and more impaired touch discrimination at one and six months post stroke. Our results extend these prior findings by showing that already at one week after stroke, the extent of altered (reduced) inter-hemispheric functional connectivity of the somatosensory network is associated with more severe impairments in somatosensory function. Together, these observations highlight the behavioural relevance of the integrity of resting-state inter-hemispheric connectivity for both motor and somatosensory function and therefore provide further support to the notion that assessments of restorations/normalizations of these altered interhemispheric connectivity patterns may form a reliable index for evaluating the effectiveness of rehabilitation therapies at the neural level [6, 17, 52].

#### 4.1.2 Intrahemispheric functional connectivity

In general, decreased ipsilesional and increased contralesional activity is described and associated with impaired function [12, 25, 53–56]. Albeit, several different effects are described for the acute phase. A paper of Rehme et al [14] included increased intrahemispheric functional connectivity in their criteria for classification of upper limb function. In contrast, several studies showed the association between decreased ipsilesional intrahemispheric functional connectivity and impaired sensorimotor function [16, 57]. For example, a longitudinal study of Nijboer and colleagues [57] reported a significant lower functional connectivity between motor areas within the lesioned hemisphere compared to the contralesional hemisphere at five weeks post stroke. Also Park et al. [16] reported a decrease in ipsilesional intrahemispheric functional connectivity between M1 and the sensorimotor cortex at one week post stroke. However, this group also reported increased intrahemispheric functional connectivity between ipsilesional M1 and ipsilesional cerebellum and thalamus [16]. In our results and as anticipated, higher ipsilesional intrahemispheric connectivity was found for the group with mild to moderate somatosensory impairments. On the other hand, and perhaps surprising, functional connectivity within the contralesional hemisphere did not show any significant associations with somatosensory function, neither in the analysis between severe and mild to moderate patients, nor in the nonparametric correlations with different somatosensory modalities. Explanations for this non-conforming finding can be our sample size or lesion location or size of our subjects. For example, in a rat model, only increased functional connectivity was found in rats with extensive lesions [50]. So far, in contrast to knowledge about motor function, literature did not report explicit findings for somatosensory function and associated changes in intrahemispheric functional connectivity. Only the study of Bannister et al. [24] reported alterations in intrahemispheric connectivity in patients with stroke, but it was not specified whether this was different from healthy people or in which direction the alterations were seen. Our results of ipsilesional intrahemispheric functional connectivity were in line with previous studies for motor function. However, we are not aware of studies explicitly reporting associations of somatosensory function with intrahemispheric connectivity.

#### 4.1.3 Associations with somatosensory function

As hypothesized, we found strong associations between interhemispheric network connectivity indices and somatosensory assessments with significant correlations for PTT, light touch and stereognosis.

As shown in Fig 4, the associations with PTT and light touch were mainly driven by group (mild to moderate vs severe impaired somatosensory function) in contrast to stereognosis, for which correlations remain significant after correction for group. Thus, for PTT and light touch there were rather two groups of impairment levels than a continuum of impairment severity. First, moderate significant correlations were found between the perceptual threshold of touch (PTT) and the index of ipsilesional intra- and interhemispheric functional connectivity. PTT is a standardized measure of light touch performance, by applying transcutaneous electrical nerve stimulation (TENS) on the light touch receptors of the skin, and activating the large myelinated Aβ fibres [33]. In previous work of our research group, higher prevalence of light touch deficits was reported based on PTT compared to other assessment methods [38], highlighting the further relevance of this assessment method for future research when investigating upper limb somatosensory deficits.

The association between stereognosis and interhemispheric connectivity can be explained by the integrative properties of stereognosis. Somatosensory information from the arm and hand will be integrated to obtain information of different properties of the object, such as size, shape, and weight. [58]. Thus, the integration of these properties combined with proprioceptive information could explain the significant correlation between stereognosis and functional connectivity [59]. Our findings therefore extend previous literature by Borstad and colleagues [60], who reported the association between haptic performance and the integrity of interhemispheric tracts connecting bilateral frontoparietal white matter and M1 in chronic stroke patients. As we found strong associations between interhemispheric network connectivity indices and stereognosis.

### 4.2 Strengths and limitations

Our study investigated 19 patients in the acute phase post stroke. The testing protocol consisted of state-of-the-art clinical and instrumented assessments for somatosensory function and a resting-state fMRI scan. One of the strengths of this study is that we used an extended and standardised test battery to investigate several somatosensory modalities of the included patients. A second strength is the use of resting state fMRI to be able to include and investigate severely impaired patients. However, a few limitations need to be considered. First, only the affected upper limb of the patients was assessed. Due to the extended test battery and the limited work load of patients in the acute phase, we chose to not assess the contralateral upper limb in addition. Second, the effect of the lesion area on the connectivity data is unknown. To date, different methods have been adopted to correct for this influence. One way to consider the effects is to include a cost-function modification excluding the lesion area during spatial normalization [61, 62]. In the current study, we have remediated this possible bias by excluding the ROI’s located into the lesion area [11]. Third, there was no group of healthy subjects included; therefore, a comparison to healthy functional connectivity in these brain areas was prohibited. However, we have been able to demonstrate differences in somatosensory networks within the acute stroke population, namely according to somatosensory severity. Another limitation relates to our relatively small sample. Although this was the first study conducted in this population with a focus on somatosensory function early after stroke, it would be worthwhile to replicate our results within a larger sample and with the addition of healthy controls. Further, resting-state fMRI is based on fluctuations in BOLD signal, which can be influenced by haemodynamic characteristics. Potential sources of noise such as cardiac- and respiratory-related variables are reported to possibly confound the interpretation of resting state data in healthy subjects [63, 64]. However, our inclusion of mean framewise displacement as a variable of no interest limits the possible confounding by movement. Further research to investigate the possible influence of stroke and stroke types on haemodynamic parameters is recommended.

With this study, we investigated the differences in functional connectivity in the acute phase post stroke associated with somatosensory impairments in the upper limb. We found that higher connectivity was associated with better somatosensory function at one week. Further research should address stroke-related changes over time in functional connectivity and their relationship with presence and recovery of somatosensory deficits. In addition, longitudinal changes in functional connectivity related to the effects of a somatosensory rehabilitation program and the association with recovery, need to be investigated.

## Conclusion

This resting-state fMRI study investigated the somatosensory network in the acute phase after stroke. The results provide evidence that the somatosensory network is a vulnerable network to be associated with somatosensory deficits after stroke. This study showed that higher functional network connectivity in ipsilesional intra- and interhemispherical network indices were related to better somatosensory function. Furthermore, the association between brain connectivity and somatosensory function was most pronounced for light touch function. The novelty of this study is the use of resting state fMRI in combination with an extended test battery of standardized clinical assessments and a more sensitive measurement, i.e. the perceptual threshold of touch to investigate the relationship between somatosensory deficits and functional connectivity of the brain in the acute phase post stroke. To the best of our knowledge, the differences in functional connectivity between severe and mild to moderate somatosensory impairments was not investigated yet. Further research is needed to confirm these results and to gain further insights in brain connectivity related to somatosensory function throughout the different phases and related to recovery and therapy post stroke, including studying differences with healthy control subjects. To this end, knowledge about the underlying neural connectivity change post stroke might be valuable for prognosis and therapy selection in future.

## Supporting information

S1 Table Prevalence of ROI's that showed overlap with the lesions of each patient. ROI: region of interest.
(DOCX)

S2 Table Overview of lesion location for each patient. ACM: arteria cerebri media; ACA: arteria cerebri anterior; ACP: arteria cerebri posterior; BA: arteria basilaris; C: cortical lesion; SC: subcortical lesion.
(DOCX)
