# Supplementary material for: Functional network connectivity is altered in patients with upper limb somatosensory impairments in the acute phase post stroke: A cross-sectional study
Source: PLoS One. 2018 Oct 12;13(10):e0205693. doi: 10.1371/journal.pone.0205693 (PMC6185852; doi:10.1371/journal.pone.0205693)
Supplement: S1 Table — ROI: region of interest. (DOCX) [file pone.0205693.s001.docx]

**Inline Supplementary Table 1. Prevalence of ROI's that showed overlap with the lesions of each patient.** *ROI: region of interest*

|  |  | **LEFT** n (%) | **RIGHT** n (%) |
| --- | --- | --- | --- |
| Primary sensorimotor cortex | **SM1** | 0 | 6 (31.58) |
| Superior parietal cortex | **SPC** | 0 | 2 (10.53) |
| Supplementary motor cortex | **SMA** | 0 | 0 |
| Inferior parietal cortex | **IPC** | 0 | 6 (31.58) |
| Insula | **INS** | 2 (10.53) | 11 (57.89) |
| Cerebellum | **CER** | 0 | 0 |
| ROI: region of interest | | |  |
