# Supplementary material for: Functional network connectivity is altered in patients with upper limb somatosensory impairments in the acute phase post stroke: A cross-sectional study
Source: PLoS One. 2018 Oct 12;13(10):e0205693. doi: 10.1371/journal.pone.0205693 (PMC6185852; doi:10.1371/journal.pone.0205693)
Supplement: S2 Table — ACM: arteria cerebri media; ACA: arteria cerebri anterior; ACP: arteria cerebri posterior; BA: arteria basilaris; C: cortical lesion; SC: subcortical lesion. (DOCX) [file pone.0205693.s002.docx]

| **Inline Supplementary Table 2. Overview of lesion location for each patient** | | | | | |
| --- | --- | --- | --- | --- | --- |
| **Subject** | **Type of stroke** | **Side of lesion** | **Cortical (C) or subcortical (SC)** | **Location of lesion** | **Group allocation** |
| 1 | ischemic | right | C + SC | ACM | Mild to moderate |
| 2 | haemorrhagic | right | C + SC | ACM | Mild to moderate |
| 3 | ischemic | right | C + SC | ACM | Mild to moderate |
| 4 | haemorrhagic | right | C + SC | ACM | Mild to moderate |
| 5 | ischemic | right | C + SC | ACM | Severe |
| 6 | ischemic | right | C + SC | ACA | Mild to moderate |
| 7 | ischemic | right | C + SC | ACM | Mild to moderate |
| 8 | haemorrhagic | right | C + SC | ACM | Severe |
| 9 | ischemic | right | C + SC | ACM | Severe |
| 10 | ischemic | left | SC | ACM | Severe |
| 11 | ischemic | right | C + SC | ACM | Mild to moderate |
| 12 | ischemic | right | C + SC | ACM | Severe |
| 13 | ischemic | left | brainstem | BA | Mild to moderate |
| 14 | ischemic | left | brainstem | BA | Mild to moderate |
| 15 | haemorrhagic | right | C + SC | ACM + ACP | Mild to moderate |
| 16 | ischemic | right | C + SC | ACM | Severe |
| 17 | ischemic | right | C + SC | ACM | Severe |
| 18 | ischemic | left | C + SC | ACM | Severe |
| 19 | haemorrhagic | right | C + SC | ACM + ACP | Severe |
| ACM: arteria cerebri media; ACA: arteria cerebri anterior; ACP: arteria cerebri posterior; BA: arteria basilaris; C: cortical lesion; SC: subcortical lesion | | | | |  |
